# Rapid large-scale preparation of ZnO nanowires for photocatalytic application

**DOI:** 10.1186/1556-276X-6-536

**Published:** 2011-10-03

**Authors:** Chunyu Ma, Zhihua Zhou, Hao Wei, Zhi Yang, Zhiming Wang, Yafei Zhang

**Affiliations:** 1 Key Laboratory for Thin Film and Microfabrication of Ministry of Education, Research Institute of Micro/Nano Science and Technology, Shanghai Jiao tong University, Shanghai 200240, China; 2 State Key Laboratory of Electronic Thin Film and Integrated Devices, University of Electronic Science and Technology of China, Chengdu 610054, China

## Abstract

ZnO nanowires are a promising nanomaterial for applications in the fields of photocatalysis, nano-optoelectronics, and reinforced composite materials. However, the challenge of producing large-scale ZnO nanowires has stunted the development and practical utilization of ZnO nanowires. In this study, a modified carbothermal reduction method for preparing large-scale ZnO nanowires in less than 5 min is reported. The preparation was performed in a quartz tube furnace at atmospheric pressure without using any catalysts. A mixed gas of air and N_2 _with a volume ratio of 45:1 was used as the reactive and carrier gas. About 0.8 g ZnO nanowires was obtained using 1 g ZnO and 1 g graphite powder as source materials. The obtained nanowires exhibited a hexagonal wurtzite crystal structure with an average diameter of about 33 nm. Good photocatalytic activity of the nanowires toward the photodegradation of methylene blue dye under UV irradiation was also demonstrated.

## Introduction

Organic dyes widely used in rubber, textiles, and plastics industries are one of the largest groups of pollutants released into wastewaters [1]. They have caused severe environmental contamination because of potential toxicity of the dyes and their visibility in water bodies. Degradation and removal of them are a vital matter for protecting the environment. However, the traditional techniques for treating organic dyes are usually non-destructive, ineffective, and costly or just transfer pollutions from water to another phase [2].

Recently, it has been reported that ZnO can be an alternative to conventional treatments for removing dye pollutants from water [3]. ZnO, with a lower cost, absorbs over a larger fraction of UV spectrum and absorbs more light quanta than TiO_2 _[4]. When an appropriate light source illuminates ZnO, electron/hole pairs will be produced with electrons absorbing the light energy, transitioning to the conduction band and leaving positive holes in the valence band [5]. The produced electron/hole pairs induce a complex series of reactions that might lead to the complete degradation of the dye pollutants adsorbed on the semiconductor surface. Specifically, ZnO nanowires have demonstrated excellent photocatalytic activity because of their larger surface area and higher surface state [6].

Until now, there are various techniques, such as chemical vapor deposition [7], physical vapor deposition [8], electrodeposition [9,10], and thermal evaporation [11] that can be used to synthesize ZnO nanowires. These methods have made great contribution to the development of ZnO-based nanoelectronic devices, such as solar cells [12], light-emitting diodes [8], field-effect transistors [13], field-emission displays [14], and biosensors [15]. However, for employing ZnO nanowires as photocatalysts, massive ZnO nanowires are needed for practical utilization. Thus, it is necessary to develop a method to fabricate large-scale ZnO nanowires.

In this report, a modified carbothermal reduction method was developed and employed to fabricate large-scale ZnO nanowires. The fabrication process took less than 5 min. The surface morphology, microstructure, crystal structure, and optical properties of the prepared ZnO nanowires were characterized. In addition, the photocatalytic activity of the ZnO nanowires was also evaluated using methylene blue (MB) as a model dye.

## Experimental

### Preparation of ZnO nanowires

A quartz tube furnace with an inner diameter of 7.5 cm was first heated to 1150°C. Then, the furnace was purged with a mixed gas of air (0.1 L/min) and N_2 _gas (4.5 L/min) continuously. Subsequently, mixtures of ZnO (Sinopharm Chemical Reagent Co., Ltd) and graphite (325 mesh) powder (2 g) with a weight ratio of 1:1, which was grounded in an agate mortar beforehand, were transferred to a quartz boat, and then the boat was placed in the middle of the furnace. After about 1 min, white snowflake-like product was carried out by the carrier gas. A 5000-mL flask, covered at the downstream of the quartz tube furnace, was used to collect the prepared product. The total reaction time was about 5 min. A white layer of products was deposited on the inner surface of the flask lastly. The weight of the obtained ZnO nanowires was about 0.8 g, which is 80% of the source ZnO powder.

### Sample characterizations

The crystal structure of the prepared ZnO nanowires was analyzed using X-ray diffraction (XRD, D/max-2200/PC, Rigaku). The morphology and microstructure of the nanowires were characterized using scanning electron microscopy (SEM, Ultra 55, Carl Zeiss) and transmission electron microscopy (TEM, JEM-2100, JEOL). The chemical composition of the nanowires was analyzed by the energy dispersive X-rays spectroscopy (EDS) with the SEM. The Raman signals of the ZnO nanowires were recorded using an Ar^+ ^ion laser as the excitation (514.5 nm) at room temperature (French Labrum-HR). The photoluminescence (PL) spectra of the ZnO nanowires were performed using a He-Cd laser line of 325 nm as an excitation source (Jobin Yvon LabRAM HR 800UV).

### Photocatalytic studies

The photocatalytic activity of the prepared ZnO nanowires was studied by using MB as a model dye. The prepared ZnO nanowires (20 mg) were dispersed in 100 mL of MB aqueous solution (10 mg/L). Before irradiating, the above mixture solution was sonicated for 30 min for establishing absorption-desorption equilibrium. A 1000 W xenon lamp was used as the light source, and placed at about 120 cm above of the mixture solution. The intensity of UV part between the wavelengths range of 320-400 nm was measured to be about 8 mW/cm^2^. The experiments were performed for 60 min, and samples were taken every other 10 min. The concentration of MB was monitored by measuring the absorbance of the supernatant at 665 nm using a UNICO UV-2102 spectrometer. A control test of MB photodegradation in absence of ZnO nanowires was also performed.

## Results and discussion

**Figure 1:**
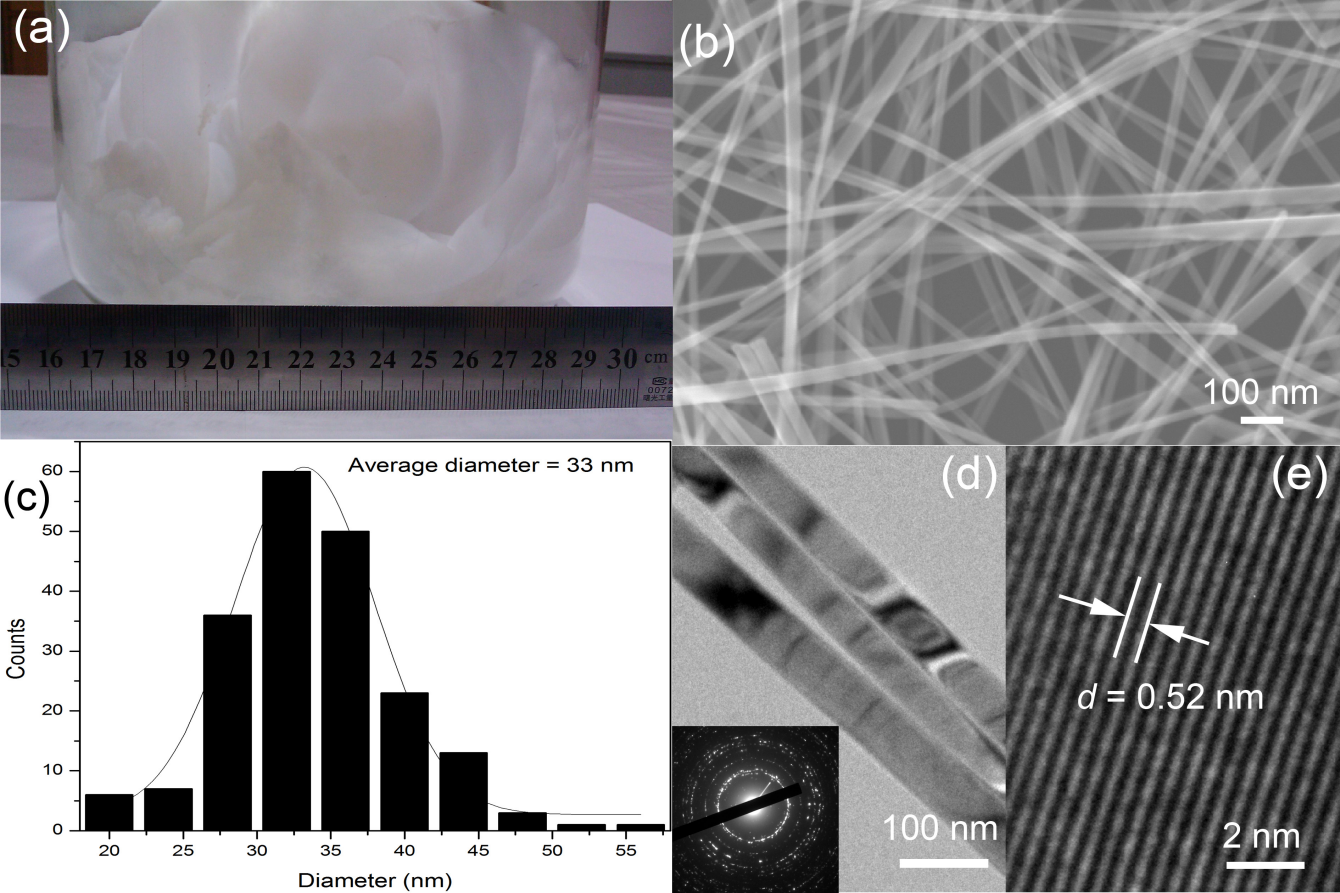
**Morphology characterization of the prepared ZnO nanowires**. **At (a) **photographic image, **(b) **typical SEM image, **(c) **the diameter distribution histograms, **(d) **TEM image, and (e) HRTEM image. Inset is a typical electron diffraction pattern of a single ZnO nanowire.

Figure 1a shows a photographic image of the prepared ZnO nanowires collected in a 5000-mL flask using the mixture powder with 2.0 g as a source material. The ZnO nanowires formed a white cotton-like semi-transparent thin film on the inner wall of the flask. A high yield (0.8 g ZnO nanowires) of the product was achieved. Figure 1b presents a typical SEM image of the ZnO nanowires. It shows a general view of the morphology of the nanowires. The ZnO nanowires are observed as entangled and curved wire-like structure. The edges of the ZnO nanowires are smooth.

Size uniformity is an important index to evaluate the quality of nanomaterials. Here, the size uniformity of the prepared ZnO nanowires was investigated by studying the diameter distribution of the nanowires. The diameters of the prepared ZnO nanowires were extracted from 200 nanowires in several SEM images. The diameter distribution histogram is shown in Figure 1c. It can be seen that the diameters of most of the nanowires ranged from 30 to 40 nm. The black solid line is the corresponding Gaussian line-fitting. It shows that the average diameter of these nanowires was about 33 nm.

The detailed microstructure of the ZnO nanowires was further characterized using TEM. Figure 1d shows a typical low-magnification TEM image of the prepared ZnO nanowires, revealing the representative morphology of the ZnO nanowires. It shows several straight and smooth nanowires with no secondary growth or extra structural features. Inset in Figure 1d shows a typical electron diffraction pattern of a single ZnO nanowire, suggesting the polycrystalline nature of the nanowires. An HRTEM image taken from a single nanowire is presented in Figure 1e. The clear lattice fringes in the HRTEM image indicate that the nanowire had a high degree of crystallinity and no amorphous materials existed at the interface. The lattice spacing is measured to be approximately 0.52 nm, which agrees well with the spacing of the (002) planes of the wurtzite ZnO structure. The TEM results suggest that the ZnO nanowires were structurally uniform polycrystalline nanowires.

**Figure 2:**
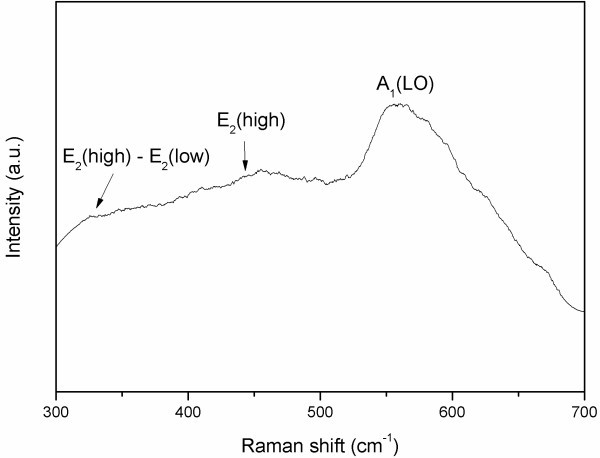
**Raman spectrum of the prepared ZnO nanowires**.

Raman scattering is sensitive to the microstructure of nanomaterials. It was used here to further study the structure of the ZnO nanowires. Figure 2 shows a typical room temperature Raman spectrum of the ZnO nanowires. The spectrum exhibited two usual modes in ZnO, including 333 (*E*_2_(high)-*E*_2_(low) [16]) and 437 cm^-1 ^(*E*_2_(high)), which is relatively weak in intensity [17]. Another noticeably A_1_(LO) mode at about 573 cm^-1 ^[18] is also observed, which is attributed to the electric field-induced Raman scattering [19], revealing the high quality of the ZnO nanowires.

**Figure 3:**
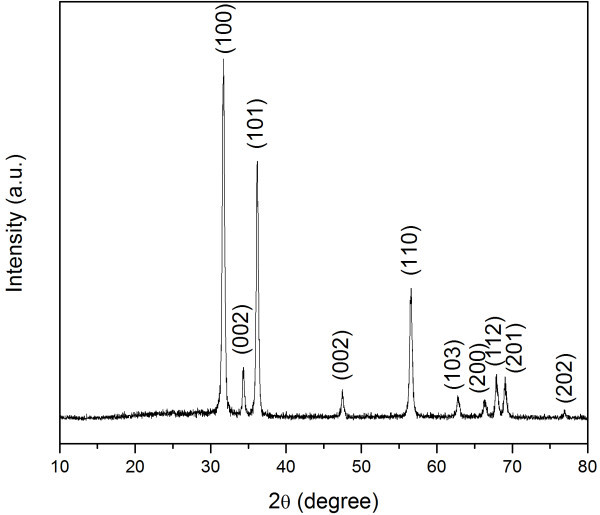
**XRD pattern of the prepared ZnO nanowires**.

The crystal structure of the ZnO nanowires was measured by XRD analysis. Figure 3 shows a typical XRD pattern of the ZnO nanowires recorded from 10°to 80°. The diffraction peaks are exactly indexed to the hexagonal wurtzite ZnO phase (JCPDS 65-3411) with cell constants of *a *= 3.24 Å and *c *= 5.19 Å. Diffraction peaks associated with zinc or carbon were not detected in the prepared sample. The broadening of ZnO peaks is due to the small nanowire size.

**Figure 4:**
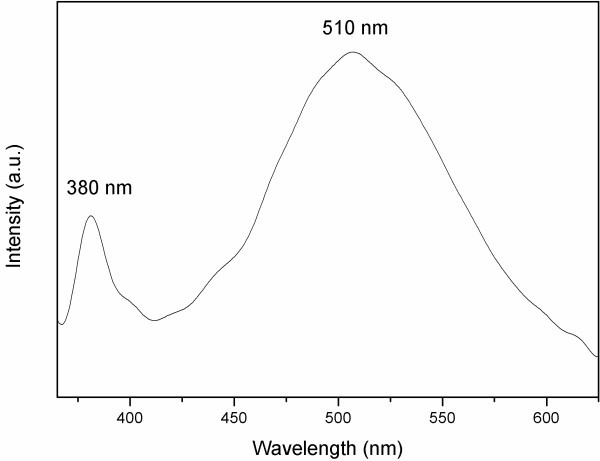
**Room temperature photoluminescence spectrum of the prepared ZnO nanowires excited at 325 nm**.

Optical properties of functional nanomaterials are of importance considering further applications in nanoelectronic devices [20]. Therefore, the optical properties of the prepared ZnO nanowires were further investigated by PL spectroscopy. Figure 4 presents the PL spectrum of the prepared ZnO nanowires. Two emitting bands, including a strong ultraviolet emission at about 386 nm and a very week green band (450-550 nm), were observed. The strong ultraviolet emission is contributed to the near band edge emission of the wide bandgap ZnO. The green band emission is due to the singly ionized oxygen vacancy in ZnO and is an effect of the recombination of a photogenerated hole with the single ionized charge state of defects [21]. The intensity of the green luminescence is proportional to the amount of singly ionized oxygen vacancies. Here, the almost negligible green band indicates that there is a very low concentration of oxygen vacancies in the prepared ZnO nanowires.

**Figure 5:**
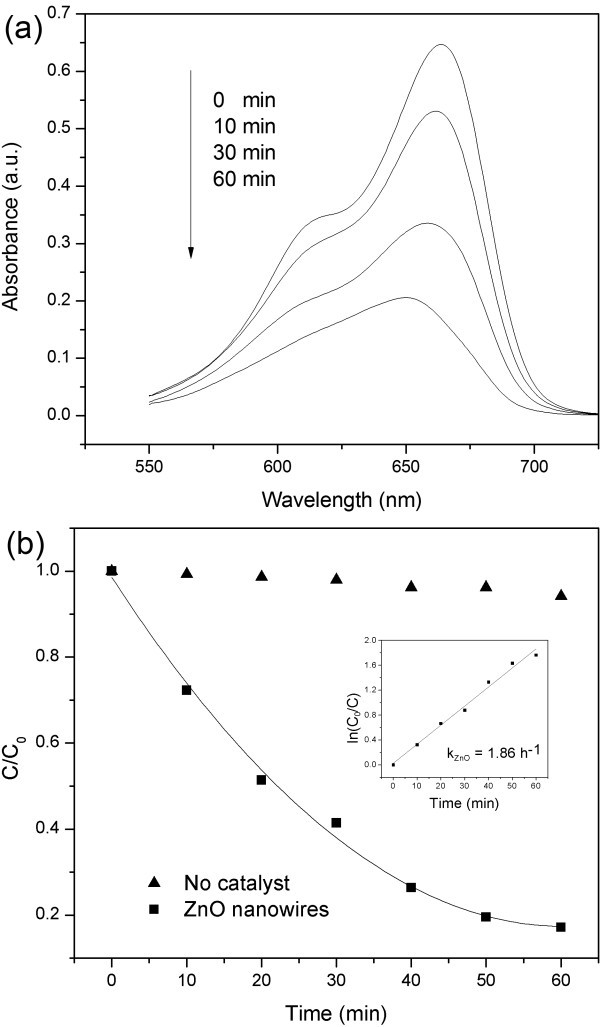
**Photocatalytic activity of the ZnO nanowires toward the photodegradation of methylene blue (MB)**. **At (a) **Time-dependent UV-Vis absorbance spectra of the MB solution using the prepared ZnO nanowires as photocatalysts and **(b) **Curves of the degradation rate of the MB dye and UV irradiation time with and without the photocatalyst of the prepared ZnO nanowires (*C*_0 _is the initial concentration of MB, *C *is the reaction concentration of MB at time *t*). Inset is the ln(*C*_0_/*C*) versus time curve of photodegradation of MB in the presence of the prepared ZnO nanowires.

ZnO has been considered to be a promising photocatalysts for the degradation of organic contaminant [2,22,23]. The photocatalytic activity of the ZnO nanowires was evaluated using MB as a model dye. Figure 5a shows the time-dependent absorption spectra of MB degradation over ZnO nanowires under UV irradiation. It can be seen that the absorbance peak at 665 nm is reduced significantly, which indicated the degradation of MB molecules. Figure 5b shows curves of MB degraded with and without using the prepared ZnO nanowires as photocatalysts. It was found that the self-degradation of MB without using photocatalysts can be neglected. The concentration of MB decreased gradually with increasing exposure time in the presence of ZnO nanowires. Inset in Figure 5b shows the corresponding ln(*C*_0_/*C*) versus time curve. The curve shows a linear relationship with the irradiation time, indicating that the photodegradation of MB over the prepared ZnO nanowires proceeded through the pseudo-first-order kinetic reaction. The value of *k*, which is the photodegradation rate constant, was fitted to be about 1.86 h^-1^.

## Conclusions

In summary, we have demonstrated a rapid and catalyst-free technique for preparing large-scale ZnO nanowires. The technique is based on a modified carbothermal reduction method. ZnO nanowires prepared by this technique had a hexagonal wurtzite crystal structure, with an average diameter of 33.5 nm. Microstructure and optical properties characterization results indicated that the ZnO nanowires had a polycrystalline structure with a low concentration of oxygen vacancies. Photocatalytic activity evaluation measurements showed that the ZnO nanowires had a photodegradation rate constant of 1.86 h^-1 ^to MB dye under UV irradiation.

## Competing interests

The authors declare that they have no competing interests.

## Authors' contributions

CYM prepared the manuscript and participated in measurements. ZHZ performed the experiment. ZY helped in technical support for experiments. HW participated in measurements. ZMW provided useful suggestions. YFZ supervised all of the study. All the authors discussed the results and approved the final manuscript.
